# Abnormal Intrinsic Brain Activity and Neuroimaging-Based fMRI Classification in Patients With Herpes Zoster and Postherpetic Neuralgia

**DOI:** 10.3389/fneur.2020.532110

**Published:** 2020-10-22

**Authors:** Jiabin Huang, Yongxin Li, Huijun Xie, Shaomin Yang, Changyu Jiang, Wuping Sun, Disen Li, Yuliang Liao, Xiyuan Ba, Lizu Xiao

**Affiliations:** ^1^Department of Pain Medicine and Shenzhen Municipal Key Laboratory for Pain Medicine, Huazhong University of Science and Technology Union Shenzhen Hospital, Shenzhen, China; ^2^Formula-Pattern Research Center, School of Traditional Chinese Medicine, Jinan University, Guangzhou, China

**Keywords:** herpes zoster, postherpetic neuralgia, neuropathic pain, resting-state fMRI, aptitude of low-frequency fluctuation (ALFF), support vector machine

## Abstract

**Objective:** Neuroimaging studies on neuropathic pain have discovered abnormalities in brain structure and function. However, the brain pattern changes from herpes zoster (HZ) to postherpetic neuralgia (PHN) remain unclear. The present study aimed to compare the brain activity between HZ and PHN patients and explore the potential neural mechanisms underlying cognitive impairment in neuropathic pain patients.

**Methods:** Resting-state functional magnetic resonance imaging (MRI) was carried out among 28 right-handed HZ patients, 24 right-handed PHN patients, and 20 healthy controls (HC), using a 3T MRI system. The amplitude of low-frequency fluctuation (ALFF) was analyzed to detect the brain activity of the patients. Correlations between ALFF and clinical pain scales were assessed in two groups of patients. Differences in brain activity between groups were examined and used in a support vector machine (SVM) algorithm for the subjects' classification.

**Results:** Spontaneous brain activity was reduced in both patient groups. Compared with HC, patients from both groups had decreased ALFF in the precuneus, posterior cingulate cortex, and middle temporal gyrus. Meanwhile, the neural activities of angular gyrus and middle frontal gyrus were lowered in HZ and PHN patients, respectively. Reduced ALFF in these regions was associated with clinical pain scales in PHN patients only. Using SVM algorithm, the decreased brain activity in these regions allowed for the classification of neuropathic pain patients (HZ and PHN) and HC. Moreover, HZ and PHN patients are also roughly classified by the same model.

**Conclusion:** Our study indicated that mean ALFF values in these pain-related regions can be used as a functional MRI-based biomarker for the classification of subjects with different pain conditions. Altered brain activity might contribute to PHN-induced pain.

## Introduction

Herpes zoster (HZ) is one of the leading causes of severe pain in China, with a prevalence estimated to be 7.7% of the population who seek medical care. Moreover, 29.8% of HZ patients may develop postherpetic neuralgia (PHN) that causes pain for months or even years (1). Neuropathic pain is well-understood to have a negative impact on the quality of life and a significant impact on cognitive function, including attention, memory, and executive functions (2). Previous studies have shown that peripheral neuropathic pain arises from injury of the peripheral and the central nervous systems (3–5). HZ is caused by the reactivation of varicella zoster virus and produces typical neuropathic pain. It can be classified as HZ and PHN. HZ is characterized by a painful erythematous rash in the affected dermatome (6). PHN is a prototypical human chronic neuropathic condition exhibiting multiple signs of peripheral and central neuropathy (7). The clinical manifestations include burning, tingling, hyperesthesia, and allodynia in the affected dermatome.

Although previous studies have shown that neuropathic pain can change brain plasticity (8), the basis of the brain structural and functional changes in patients with neuropathic pain is not clear. Previous neuroimaging studies have reported that patients with PHN showed anatomical changes in the bilateral insula, superior temporal gyrus, left middle frontal gyrus, and right thalamus (9). PHN's effects on brain activity have been studied by resting-state functional magnetic resonance imaging (MRI) and task-functional MRI. Abnormal functional activation and intrinsic activity were detected in regions including the thalamus, insula, somatosensory, putamen, amygdala, brainstem, prefrontal lobe, and cerebellum (10–12). Quantitative cerebral blood flow (CBF) mapping in PHN patients showed significantly increased CBF values in the striatum, thalamus, insula, amygdala, and primary somatosensory cortex and decreased CBF values in the frontal cortex (5). The functional connectivity between these pain-related regions and the notable connections between the putamen and other brain regions were altered in PHN patients (13). A negative correlation was found between PHN patients' pain scores and intrinsic activity in the prefrontal cortex (14). These works indicated that the functional connectivity between the prefrontal regions and other cortical regions was modulated by pain intensity. Additionally, a graph–theoretic approach was used to calculate the small-world network alterations in PHN patients. The PHN patients exhibited decreased local efficiency and significant changes of regional nodal efficiency in the postcentral gyrus, inferior parietal gyrus, thalamus, para-hippocampus, and putamen (15). All the above studies indicate that chronic pain in PHN patients would modulate the activity and the connectivity of these pain-related regions.

The vast majority of these previous neuroimaging studies were restricted to PHN patients. Only a few studies have used neuroimaging methods to explore the differences in brain activity between HZ and PHN (11, 16). A previous study identified that HZ patients showed significant functional changes in the cerebellum, occipital lobe, temporal lobe, parietal lobe, and limbic lobe in contrast to PHN patients (11). Compared with the healthy controls (HC), both HZ and PHN patients demonstrated significantly decreased functional connectivity density (FCD) in the precuneus. However, there is no significant difference in the FCD values between HZ and PHN patients (16). One recent longitudinal neuroimaging study also assessed the brain imaging changes from HZ to PHN and found that the activity of the cerebellum and frontal lobes increased and the activity of the occipital lobe and limbic lobe decreased significantly during this transition (17).

Although previous literature confirmed that the brain function of patients with HZ and PHN was changed compared with the HC, the differences in brain activity between HZ and PHN were inconsistent across these studies, and the neural mechanism is still unclear. Based on previous literature, we hypothesized that patients with HZ and PHN exhibit significant changes in spontaneous brain activity, which can be used to classify them from healthy controls. Thus, the present study aimed to explore the effects of HZ and PHN on brain activity and detect the neural mechanism underlying cognitive impairment in neuropathic pain patients. Resting-state functional MRI data were collected from all participants, and the amplitude of low-frequency fluctuation (ALFF) was calculated. The ALFF is the most common and widely used method for characterizing the dynamic properties of the neuronal processing unit (18). The correlations between clinical pain scales and spontaneous brain activity were also assessed. Additionally, to evaluate the stability of the group comparison results, imaging features with significant differences between groups were applied for classification using support vector machine (SVM).

## Methods

### Subjects

Fifty-two patients (right-handed; 28 HZ and 24 PHN) and 20 HCs (right-handed) were recruited from the Pain Medicine Department of Huazhong University of Science and Technology Union Shenzhen Hospital. Before the imaging data were collected, we explained the purpose of the study, the study procedures, and the possible risks and discomfort to all participants. Then, written confirmed consent was obtained from each participant or their companion. This study was carried out following the Declaration of Helsinki and was approved by the Ethics Committee of Huazhong University of Science and Technology Union Shenzhen Hospital. The inclusion criteria were HZ within 30 days after onset of the painful rash, dermatome below C2, age older than 50 years old, and with persistent pain [visual analog scale (VAS) ≥ 5]. All patients were diagnosed as PHN based on persistent pain (VAS ≥ 5) for more than 30 days following the initial rash caused by HZ (19). Patients who had other pain disorders were excluded. All patients underwent MRI scanning within 24 h of enrollment. For ethical consideration, all patients had taken medicines to reach tolerable pain level before imaging. The baseline treatment is 8 mg amitriptyline (Shanghai Fosun Pharmaceutical group) and 75 mg pregabalin (Pfizer).

Before MRI scanning, spontaneous pain intensity was assessed using the VAS, which measures pain intensity on a scale of 0 to 10, with 0 indicating no pain and 10 indicating the highest tolerable pain. Additionally, to identify the qualities of pain, the McGill pain questionnaire was used, including the Pain Rating Index (PRI) and the Present Pain Index (PPI). The PRI contains four subscales to evaluate the sensory, affective and evaluative, and miscellaneous aspects of pain. The PPI is a six-level pain intensity scale, including none (0), mild (1), discomforting (2), distressing (3), horrible (4), and excruciating (5) (20, 21). The exclusion criteria included any active major psychiatric illnesses, neurological illnesses, head injuries, or alcohol or drug abuse. Twenty healthy controls (all right-handed, 13 females, mean age: 63.05 ± 26.20 months) were also recruited. Detailed information can be found in Table 1.

**Table 1:** Demographic and clinical information data of the subjects.

**Characteristics**	**HZ patients (*n* = 28)**	**PHN patients (*n* = 24)**	**HC (*n* = 20)**
Gender (male/female)	12/16	15/9	7/13
Age (years, mean ± SD)	58.2 ± 13.1	67.0 ± 14.1	63.1 ± 12.2
Handedness (right/left)	28/0	24/0	20/0
Duration (days, mean ± SD)	15.7 ± 5.6	227.0 ± 72.1	/
VAS score (mean ± SD)	7.6 ± 1.3	6.7 ± 1.6	/
PRI score (mean ± SD)	12.4 ± 6.0	16.4 ± 9.2	/
PPI score (mean ± SD)	2.9 ± 1.0	3.0 ± 1.1	/

*HC, healthy controls; HZ, herpes zoster; PHN, postherpetic neuralgia; VAS, visual analog scale; PRI, pain rating index; PPI, present pain intensity; SD, standard deviation*.

### MRI Data Acquisition

All participants underwent MRI scans using a 3T Siemens scanner (MAGNETOM Skyra, Siemens, Germany) at the Huazhong University of Science and Technology Union Shenzhen Hospital, Shenzhen, China. Foam cushions were used in the scan process to reduce head translation and rotation. The resting-state functional MRI scans were obtained using an echo-planar imaging sequence with the parameters as follows: repetition time/echo time = 2,430/30 ms, field-of-view = 240 × 240 mm^2^, matrix = 64 × 64, flip angle = 90°, slice thickness = 3.6 mm, 40 interleaved axial slices, and 180 volume. High-resolution T1-weighted 3D MPRAGE images were acquired for all subjects: repetition time/echo time = 1,900/2.12 ms, field-of-view = 256 × 256 mm^2^, 320 sagittal slices, 0.6 mm slice thickness, flip angle = 9°. All the participants were instructed to lie still with their eyes closed while remaining awake. After the scan, the subjects were asked whether they remained awake during the entire scan. All acquisitions were visually inspected for the presence of imaging artifacts. None of the participants were excluded on this basis.

### Resting-State Data Preprocessing and Statistical Analysis

The resting-state functional MRI data were processed using Data Processing Assistant for Resting-State Functional MRI (DPARSF) software (http://www.restfmri.net) (22). The first 10 functional images per subject were excluded from the analysis to ensure magnetization equilibrium. The functional MRI data were then slice-timed with a reference point at the median image and realigned to the first image. A mean functional image was obtained for each participant. No translation or rotation parameters in any given data set exceeded ±2 mm or ±2°. Afterward, each participant's T1-weighted structural image was co-registered to their mean functional image and then segmented. Nine nuisance covariates (six head motion parameters, global signal, white matter signal, and cerebrospinal fluid signal) were removed. The functional images were then normalized into the standard Montreal Neurological Institute space using the T1 image unified segmentation, resampled to 3 mm, and smoothed using a 6-mm full-width at half-maximum Gaussian smoothing kernel. Linear trends were removed before the time course data from each voxel. Finally, an approach of the ALFF method was used for detecting regional signal change of spontaneous activity (23).

The ALFF for each voxel was normalized, and one-way ANOVA was used to determine whether there were any statistically significant differences in the ALFF between these groups (*p* < 0.001, cluster size = 5). *Post hoc* analyses using two-sample *t*-tests were also conducted among the three groups. The significant results of ANOVA were selected as an output mask in the following *t*-tests. A *p* < 0.05 (a minimum cluster size of five voxels) corrected by false discovery rate (FDR) correction was considered a difference between groups. Age and gender were controlled as covariates in all the above-mentioned statistical analyses.

**Table 2:** Significant differences in amplitude of low-frequency fluctuation among three groups.

**Comparisons**	**Statistical values**			**Coordinates' anatomical location**			
	**Cluster size**	***t*-value**	***p*-value**	***x***	***y***	***z***	**Region**
HC > HZ	32	4.92	0.000	9	−57	24	R precuneus
	8	4.89	0.000	−3	−63	27	L precuneus
	15	5.46	0.000	−6	−48	27	L PCC
	5	5.38	0.000	66	−6	−27	R MTG
	7	4.67	0.000	69	−36	−12	R MTG
	9	4.62	0.000	54	−51	36	R angular
HC > PHN	72	5.80	0.000	9	−48	24	R precuneus
		5.76	0.000	−6	−48	27	L PCC
		4.83	0.000	0	−63	27	L precuneus
	6	4.66	0.000	42	48	27	R MFG
	5	4.53	0.000	63	−6	−27	R MTG

The Montreal Neurological Institute coordinates and t-values for the local maxima of the centers of the voxel clusters. The threshold for significant clusters reported here was set at p < 0.05 (false discovery rate corrected, cluster size of 5).

R, right hemisphere; L, left hemisphere; HC, healthy controls; HZ, herpes zoster; PHN, postherpetic neuralgia; PCC, posterior cingulate cortex; MTG, middle temporal gyrus; MFG, middle frontal gyrus.

All the significantly different clusters from the above ALFF analyses were extracted and considered as one region-of-interest (ROI). The mean ALFF of this ROI was extracted and normalized with Fisher's z-transformed function. The associations between the transformed ALFF value and the scores of clinical pain scales were tested using Pearson's correlation. A *p* < 0.05 was considered a statistically significant correlation.

### Subjects' Classification With Support Vector Machine

A multivariate pattern analysis was used in neuroimaging data to extract patterns and to categorize individual observations into different categories (24, 25). We used a specific multivariate pattern analysis approach known as SVM to classify the patients in the present study. SVM was implemented using the Pattern Recognition for Neuroimaging Toolbox software, version 2.0 (http://www.mlnl.cs.ucl.ac.uk/pronto/). Individual resting-state functional MRI was treated as points located in a high-dimensional space defined by the ALFF values in the preprocessed images. Significant clusters of the above ALFF analyses results were extracted and considered as a mask. This mask was applied to each preprocessed functional MRI to select the normalized ALFF values as a feature in the modeling. The classifier created in the present work (i.e., HZ vs. HC, PHN vs. HC, HZ vs. PHN) is based on binary SVMs. During the cross-validation step, a “leave-one-subject-out” method was used (24, 26). The data were split into a training set consisting of the samples from all but one subject and a validation set consisting of the samples from the left-out subject. To assess the SVM's overall accuracy, this procedure was repeated for each subject pair. The classification procedure was repeated 1,000 times.

## Results

### Demographic and Clinical Features

The clinical characteristics of all the participants are shown in Table 1. All of the recruited patients in both groups suffered pain with VAS scores ≥5, indicating moderate-to-severe pain. PRI and PPI were also assessed immediately before MRI scanning in most patients (24 HZ and 20 PHN). Eight patients were not assessed for PRI and PPI because of old age and being dialect-speaking. The doctor–patient communication is not smooth, which had caused a challenge in evaluating the scale accurately.

### Between-Group ALFF Differences

**Figure 1:**
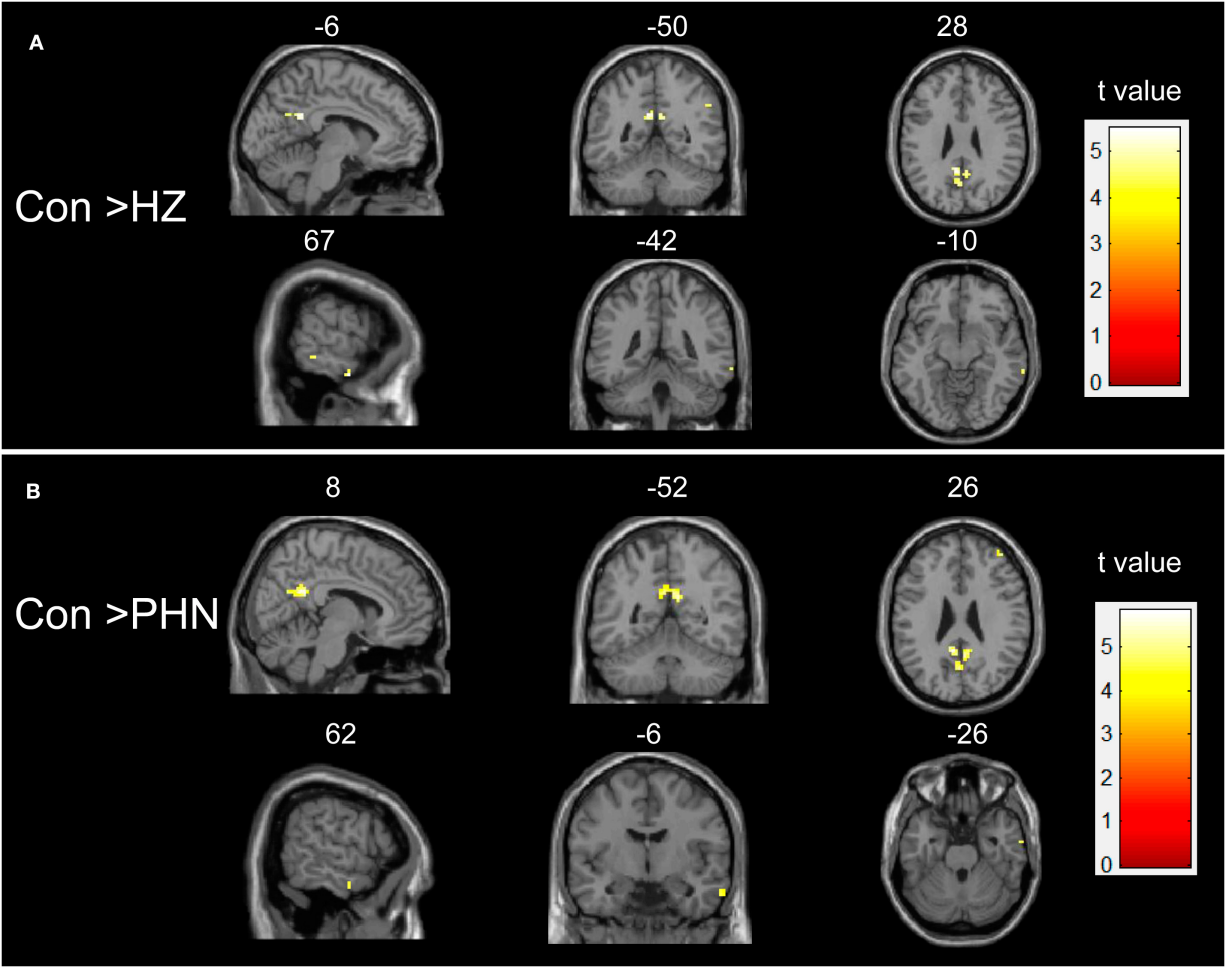
Intergroup difference in amplitude of low-frequency fluctuation (ALFF). Both comparisons of HZ vs. healthy controls (HC) **(A)** and PHN vs. HC **(B)** groups showed a significant decrease in ALFF in some brain areas. Threshold of the whole brain: *p* < 0.05, false discovery rate corrected, cluster size > 5. HZ, herpes zoster; PHN, postherpetic neuralgia; Con, normal controls.

One-way ANOVA test demonstrated that several regions showed significant differences in ALFF values among the three groups (Supplementary Figure 1). These regions included the bilateral precuneus, bilateral superior occipital gyrus, left posterior cingulate cortex (PCC), right middle frontal gyrus (MFG), right calcarine, right middle temporal gyrus (MTG), and right angular gyrus (AG). Compared with HC, patients with HZ showed decreased ALFF in the bilateral precuneus, left PCC, right MTG, and right AG (FDR corrected; Table 2, Figure 1). No region showed a significant increase of ALFF in the HZ group. In the PHN group, compared with the control subjects, a significant decrease of ALFF values was found in the bilateral precuneus, right MFG, and left PCC (FDR corrected; Table 2, Figure 1). However, the ALFF was not significantly different between the HZ and the PHN groups.

### Correlation Between ALFF and Clinical Scale

**Figure 2:**
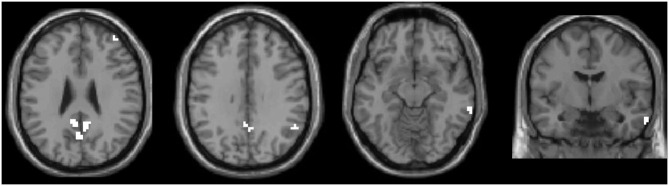
All significant clusters of the intergroup amplitude of low-frequency fluctuation analyses were extracted as one region of interest (ROI). This ROI was used in the following correlation and classification analyses.

**Figure 3:**
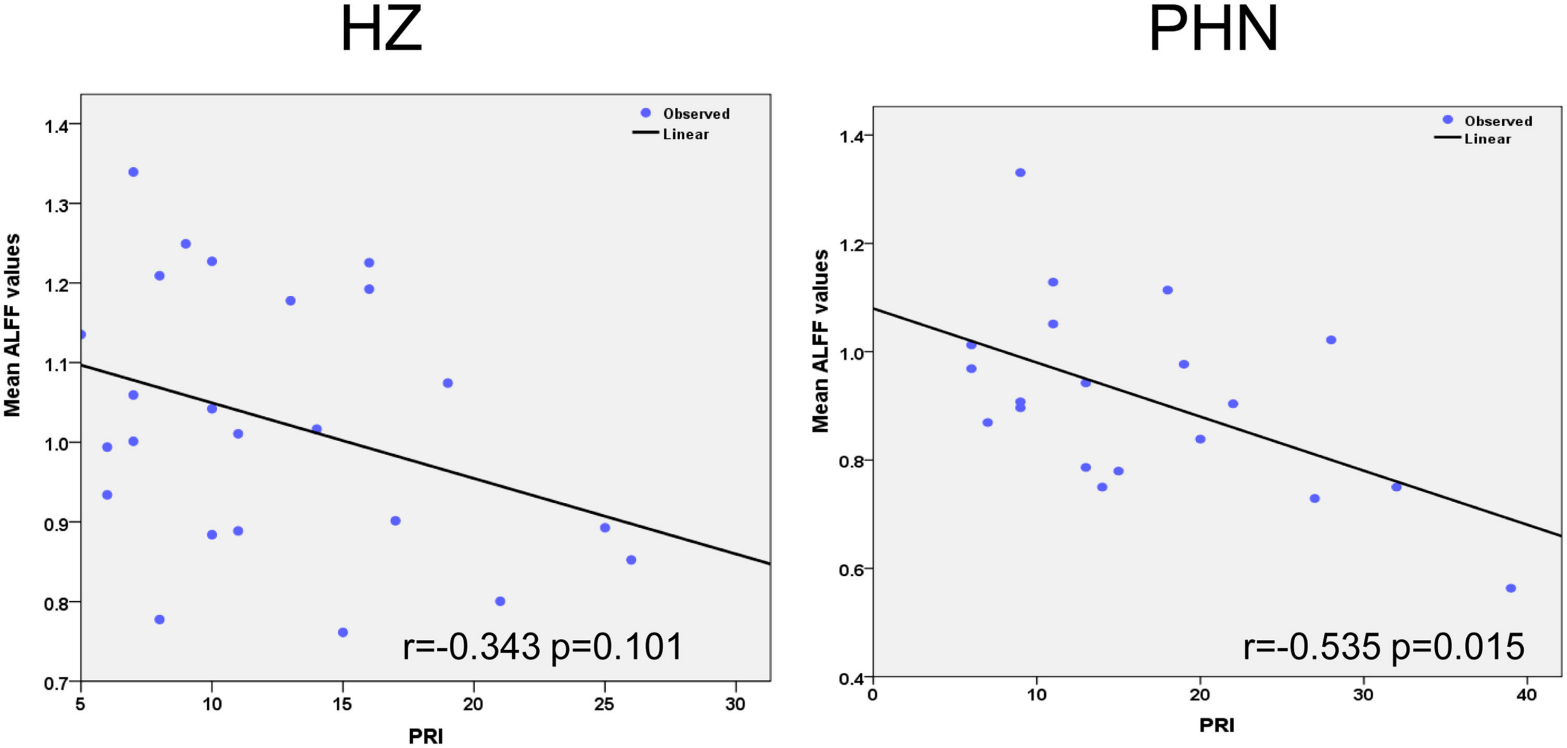
Significant correlation between the amplitude of low-frequency fluctuation and clinical variables. The left panel shows the correlation results in the herpes zoster group. The right panel showed the correlation results in the postherpetic neuralgia group.

We selected the regions showing significant changes in the above-mentioned between-group ALFF comparisons as our ROI (see Figure 2). The mean ALFF value of this ROI was extracted, and the correlations between the mean ALFF and the clinical scale were tested. There was a significant correlation between the mean ALFF and PRI in the PHN group, but not in the HZ group (PHN: *r* = −0.535, *p* = 0.015; HZ: *r* = −0.343, *p* = 0.101; see Figure 3). No significant correlation was observed between the mean ALFF and the other clinical scales.

### SVM Classification Results

**Figure 4:**
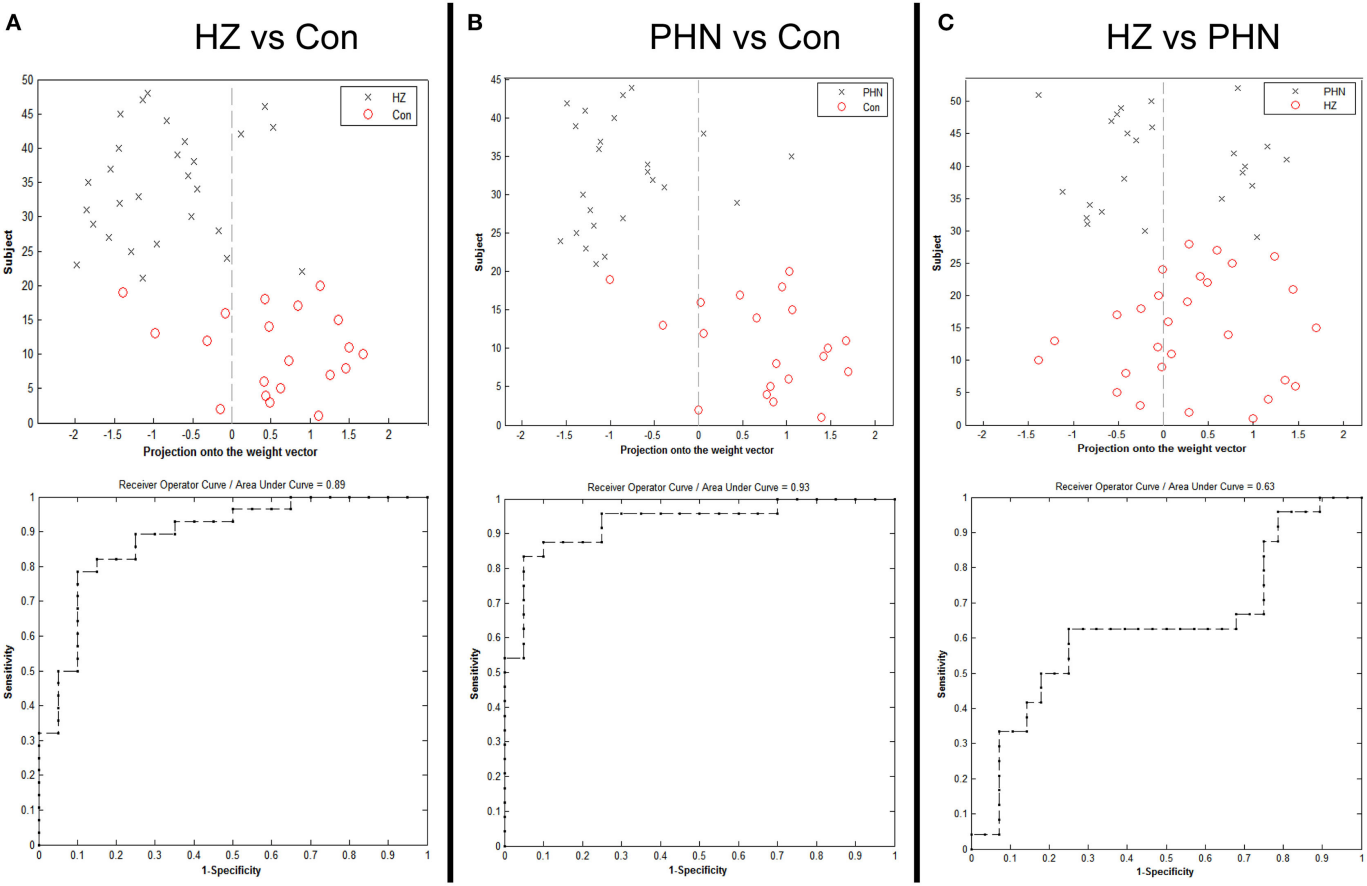
Classification plot and receiver operating characteristic curve for the comparison between the groups using mean amplitude of low-frequency fluctuation value from the region of interest. **(A)** Classification between herpes zoster (HZ) and healthy controls (HC), **(B)** classification between postherpetic neuralgia (PHN) and HC, and **(C)** classification between HZ and PHN.

ALFF in the ROI demonstrated a significant difference in patients. The SVM classification between the HZ and the HC achieved a classification accuracy of 81.25% (sensitivity 75%, specificity 85.71%, *P* < 0.001; Figure 4, left panel). Similarly, using the ALFF values of the same ROI allowed for the classification of PHN and HC subjects and achieved a classification accuracy of 86.36% (sensitivity 85%, specificity 87.5%, *P* < 0.001; Figure 4, middle panel). Finally, for HZ and PHN classification, the use of ALFF in the same ROI achieved a classification accuracy of 61.54% (sensitivity 60.71%, specificity 62.5%, *P* < 0.081; Figure 4, right panel).

## Discussion

In the present study, resting-state functional MRI and a machine learning method were combined to explore the abnormalities of spontaneous neuronal activity in patients with HZ and PHN. We demonstrated that, compared with HC, spontaneous brain activity was reduced in both patient groups. For HZ patients, reduction in neural activity was observed in the bilateral precuneus, left PCC, right MTG, and right AG. For PHN patients, the decreased activity was noted in the bilateral precuneus, left PCC, right MTG, and MFG. The mean ALFF values extracted from all these regions were significantly correlated with the clinical pain scales in the PHN group, but not in the HZ group. This result suggested that the spontaneous activity of these regions, in some respects, reflects the pain severity in PHN. In particular, machine learning classification using SVM showed that neuropathic pain patients (HZ and PHN) and healthy subjects could be classified by the decreased activity in these regions. The classifier also partially categorized patients into HZ and PHN groups (*p* < 0.081). These results may have important implications for understanding the brain abnormalities and the clinical characteristics of this disorder. Mean ALFF values in these pain-related regions may be used for the classification of neuropathic pain patients and healthy subjects.

### Significant Decrease in Spontaneous Neuronal Activity in Patients

One notable finding of this study is the significantly decreased ALFF values of the bilateral precuneus and the left PCC between groups of patients. The precuneus and the PCC are a core component of the default mode network (27). The default mode network is a network in which activity is higher than in other parts of the brain at baseline. The hub regions of this network include the bilateral PCC/precuneus, inferior parietal cortex/angular gyrus, and medial prefrontal/anterior cingulate cortex (28). The brain regions in this network are deactivated during typical cognitive load tasks, while these regions show the highest activity at baseline. Previous studies have found that PCC and the adjacent precuneus are involved in monitoring sensory information (29, 30). Information from memory and perception was combined with bottom-up attention by the precuneus/PCC. Research on brain functional connectivity of precuneus/PCC has shown disrupted connectivity in patients with PHN (16, 31). Furthermore, the precuneus has various connections with other cortical and subcortical areas, which facilitates the integration between external and internal information (32). Previous studies have also demonstrated that the precuneus plays an essential role in the implementation of a wide range of higher-order cognitive functions (33).

In the present study, we found that the ALFF values were decreased in the precuneus/PCC in the two patient groups. It has been reported that the impairment of the bilateral precuneus was associated with cognitive and behavioral impairments observed in patients with pain (34). Our finding shed light on the neural basis of increased risk of dementia in HZ and PHN patients (PMID: 29244265 and PMID: 23900759). Generally, cognitive and emotional factors have substantial impacts on pain (35). In 1979, the International Association for the Study of Pain approved a definition of pain: an unpleasant sensory and emotional experience associated with actual or potential tissue damage or described in terms of such damage (36). When actual damage is present, although the unpleasantness could rapidly disappear, the fear memory would last long (37). This result implied that the impairment of the precuneus might contribute to the emotional experience associated with pain.

In contrast to the commonly affected brain regions, decreased ALFF values in the right AG were observed only in HZ patients. The AG is an important part of the brain, which is responsible for processing language, numbers, and various logic-oriented processes. Decreased ALFF in the right AG might account for changes in the cognitive functions of HZ patients. However, no significantly decreased ALFF was found in the AG of patients with PHN. This phenomenon warrants further study in the future.

Another pain-condition-dependent brain region is MFG. In this study, a significant decrease of ALFF in the right MFG was found in patients with PHN, but not in patients with HZ. This result is consistent with previous studies that decreased ALFF value and cerebral blood flow in the MFG were detected in the patients with PHN (5, 12). The patients with PHN also showed notably abnormal tissue microstructure in the MFG (9). The right MFG has been proposed to be a site of convergence of the dorsal and the ventral attention network (38). Previous studies have identified that this region is implicated in emotion modulation and executive function (39). Therefore, our finding indicated that repressed neural activity in the MFG might be responsible for the emotional or attention aspect of PHN pain.

In addition to the precuneus/PCC, decreased ALFF values were also detected in the right MTG of both patient groups. This finding was consistent with previous studies which reported functional impairment of the right MTG in patients with PHN and HZ (11, 12, 17). However, there was no significant difference in ALFF between HZ and PHN. This result did not agree with a previous study in which PHN patients showed a decreased activity in the limbic system, right MTG, and parietal lobe compared with the HZ patients (11). We speculate that this inconsistency may arise due to the different definitions of PHN. Cao (11) defined PHN as persistent pain for more than 3 months after the zoster rash, whereas 1 month was adopted to identify PHN patients in our study (40). It is possible that this transition from HZ to PHN is gradually developed in a time-dependent fashion. A recent neuroimaging study using a similar PHN definition demonstrated that functional connectivity density was not significantly different between the PHN and the HZ patients (16). Interestingly, the results of a previous study by Hong and Cao were mildly consistent with our present study. We recently reported that temporary spinal cord stimulation could effectively prevent the development of PHN for the HZ patients (duration <3 months), compared with the PHN patients (duration >3 months) (41). Moreover, it may indicate that early neuromodulation of herpetic neuralgia could prevent the PHN effectively due to the fact that central sensitization has not yet been developed.

### Correlation Between Neuroimaging Index and the Clinical Pain Scales

More importantly, our results showed a significant correlation between the mean ALFF in the selected ROI and the clinical pain scales in the PHN group. The negative correlation suggested that the abnormal spontaneous brain activity may be due to the pain. Chronic pain condition can lead to anatomical and functional alterations in the brain regions associated with psychological modulation, resulting in not only pain but also altered cognition and affection. Cognitive behavioral therapy could reduce stress and control pain (35). Brain activity and connectivity would, in some aspects, reflect patients' cognitive ability and the pain intensity (14, 42). Chronic pain may affect the cognitive functions that depend on functional connectivity between the pain-related regions. Previous studies in brain anatomy also showed a negative correlation between brain microstructure abnormalities and PHN pain (9). Our results were consistent with these previous neuroimaging studies, which indicated that the activity of pain-related regions has potential value in objectively estimating the severity of PHN pain.

The selected ROI included regions such as the bilateral precuneus, left PCC, right MTG, right MFG, and right AG. All these regions are pain-related areas. Previous neuroimaging studies have found that the brain activity and the gray matter volume in the right MTG have a significant correlation with pain duration and intensity of patients with PHN (12, 17). The functional connectivity density of bilateral precuneus in PHN patients also showed a significant correlation with VAS scores (16). In the present study, the negative correlation results in our study indicated that the patients with more pain would show greater reduction in spontaneous brain activity in these regions. This negative correlation was not significant in the HZ group. This may be explained by the short duration of pain condition in these patients. Future studies are needed to verify this explanation.

### Identification of Patients With PHN From HC and Patients With HZ

In this study, the patients with neuropathic pain and the healthy controls were classified by the mean ALFF values of these altered regions using the linear SVM classifier. There is a high classification accuracy of 81.25% between the HZ and HC and of 86.36% between the PHN and HC. In recent years, multivariate pattern analysis methods have been applied to distinguish patients from healthy controls (43, 44) and predict surgery outcomes (25, 45). For patients with neuropathic pain, one previous neuroimaging study has found that the voxel-mirrored homotopic connectivity in the dorsolateral prefrontal cortex, precuneus, and PCC discriminated between patients with PHN and healthy subjects (31). Our recent study measuring gray matter volume found that alterations in several brain regions, including the middle frontal cortex, ACC, precuneus, and cuneus, had a significant predictive power to classify HZ patients with different responses to medications (46). Along with these findings, our observations suggested that the frontal gyrus and the precuneus have a significant predictive power to distinguish neuropathic pain patients from the healthy controls.

In the present study, we used the mean ALFF of ROI as a feature for classifying the subtypes of patients with neuropathic pain (i.e., PHN and HZ). The SVM classifier performed between HZ and PHN patients had a classification accuracy of 61.54%. We did not achieve as high a classification accuracy as in previous studies (31, 46). It should be noted that, in our study, all patients have taken pregabalin and amitriptyline to alleviate the pain intensity before the MRI scan. The previous findings revealed that amitriptyline may reduce pain-related central activations in the ACC in patients with irritable bowel syndrome (47). Similarly, pregabalin suppressed evoked neural activity in ACC, when compared with tramadol, in neuropathic pain patients (48). The current results indicated that these medicines strongly inhibited the ACC, but not the PCC, in HZ and PHN patients compared to healthy controls. Because of long-term analgesia in PHN patients clinically, it is difficult to quantify the central changes due to drug therapy or the refractory pain condition in human research.

### Limitations

There are several limitations in our study. Firstly, our present research used a cross-sectional design in which the MRI data of the subjects were obtained at just one time-point. This method may lead to questions about whether the results are the consequence of preexisting differences between groups. Future studies should consider a longitudinal design, which can directly monitor activity changes from acute pain to chronic pain. Secondly, all patients are allowed to take the drug therapy of the pregabalin and amitriptyline which may certainly inhibit or reduce the brain function. A well-controlled group should be established in further studies.

## Conclusion

In the present study, we discovered reduced spontaneous brain activity in the precuneus/PCC, MTG, AG, and MFG of patients with neuropathic pain. The reduced ALFF in these regions was associated with the clinical pain scales of patients with PHN. The SVM classification indicated that these abnormalities might accurately discriminate between neuropathic pain patients and healthy controls at the level of the individual. A similar analysis also demonstrated that the abnormalities in ALFF values showed potentials to distinguish patients with PHN from those with HZ. These results are likely to be valuable in explaining the underlying neural mechanisms and understanding the interaction between neuropathic pain and brain abnormalities. The present results provided new insights into functional MRI as a pain biomarker to help diagnose and classify patients with neuropathic pain. Future work should combine functional information with structural imaging data to examine whether this leads to a higher diagnostic accuracy.

## Data Availability Statement

All datasets generated for this study are included in the article/Supplementary Material.

## Ethics Statement

The studies involving human participants were reviewed and approved by the Ethics Committee of the Huazhong University of Science and Technology Union Shenzhen Hospital. The patients/participants provided their written informed consent to participate in this study.

## Author Contributions

WS, SY, and LX designed this work. JH, XB, DL, and YL collected the data. YL and HX analyzed the data. YL, JH, and LX discussed the results. YL wrote the initial draft. YL, JH, LX, and CJ substantially revised the manuscript. LX acquired the funding. All authors agreed to be accountable for all aspects of this work.

## Conflict of Interest

The authors declare that the research was conducted in the absence of any commercial or financial relationships that could be construed as a potential conflict of interest.

## Supplementary Material

Supplementary Figure 1 One-way ANOVA comparison on amplitude of low-frequency fluctuation maps among the three groups.
