# Supplementary figures and images for: Abnormal Intrinsic Brain Activity and Neuroimaging-Based fMRI Classification in Patients With Herpes Zoster and Postherpetic Neuralgia
Source: Front Neurol. 2020 Oct 22;11:532110. doi: 10.3389/fneur.2020.532110 (PMC7642867; doi:10.3389/fneur.2020.532110)

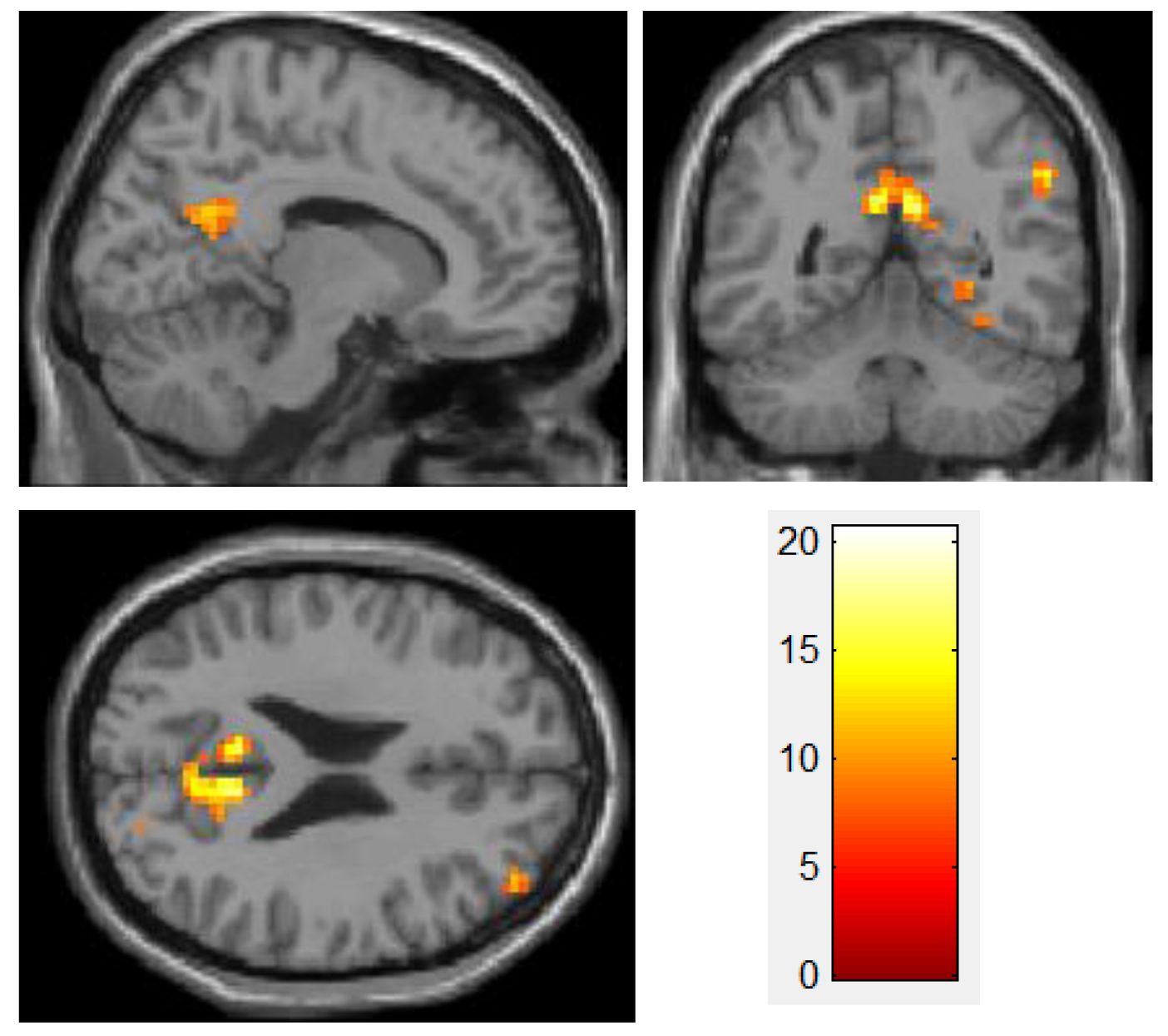

Supplement: Supplementary Figure 1 — One-way ANOVA comparison on amplitude of low-frequency fluctuation maps among the three groups. [file Image_1.TIF]
